# Influence of Age on Postoperative Neurological Outcomes after Surgery of Acute Type A Aortic Dissection

**DOI:** 10.3390/jcm10081643

**Published:** 2021-04-12

**Authors:** Mohamed Salem, Michael Salib, Christine Friedrich, Mostafa Salem, Thomas Puehler, Jan Schoettler, Felix Schoeneich, Jochen Cremer, Assad Haneya

**Affiliations:** 1 Department of Cardiovascular Surgery, School of Medicine, Christian-Albrechts-University of Kiel, Arnold-Heller-Str. 3, D-24105 Kiel, Germany; michael.salib@uksh.de (M.S.); Christine.Friedrich@uksh.de (C.F.); thomas.puehler@uksh.de (T.P.); Jan.Schoettler@uksh.de (J.S.); Felix.schoeneich@uksh.de (F.S.); Jochen.Cremer@uksh.de (J.C.); Assad.Haneya@uksh.de (A.H.); 2 Department of Cardiology and Angiology, School of Medicine, Christian-Albrechts-University of Kiel, Arnold-Heller-Str. 3, D-24105 Kiel, Germany; Mostafa.Salem@uksh.de

**Keywords:** acute type A aortic dissection, moderate hypothermic circulatory arrest, neurological complication in elderly after type A aortic dissection

## Abstract

Background: Acute type A aortic dissection (AAAD) is considered a fatal disease which requires an emergent surgical intervention. This study focuses onthe neurological outcome after surgical repair in cases of AAAD in comparison between elderly and young patients. Methods: a retrospective analysis of 368 consecutive patients who underwent emergency surgery of ascending aorta in moderate hypothermic circulatory arrest (MHCA) (20–24 °C) and antegrade cerebral perfusion after AAAD between 2001 and 2016. Patients were divided into two groups: those aged 75 years and older (68 (18.5%)) and those younger than 75 years (300 (81.5%)). Results: Comparing both groups, average age was 79.0 ± 3.2 vs. 59.2 ± 10.7 years (*p* < 0.001); female gender represents 58.8% of elderly patients vs. 28.7% in younger patients (*p* < 0.001). Intraoperatively, cardiopulmonary bypass time (155 min (131; 187) vs. 171 min (137; 220); *p* = 0.012), cross-clamping time (79 min (60; 105) vs. 93 min (71; 134); *p* = 0.001] and circulatory arrest time (29 min (22; 40) vs. 33 min (26; 49); *p* = 0.011) were significantly shorter in elderly than younger group. Postoperatively, there was no significant difference in delirium (11.8% vs. 20.5%; *p* = 0.0968) or stroke (11.8% vs. 16.1%; *p* = 0.369). The 30-day mortality was satisfactory for both groups but significantly higher in the elderly group (27.9% vs. 14.3%; *p* = 0.007). Conclusion: The current study concluded that surgical treatment of AAAD in elderly patients can be applied safely without increasing risk of neurological complication. However, minimizing operation time may help limit the occurrence of postoperative neurological complication.

## 1. Introduction

Acute type A aortic dissection (AAAD) is considered a lethal disease in the cardiovascular field, which requires emergent surgical intervention. AAAD occurs when an intimal tear arises in the innermost layer of the aorta, allowing the blood to flow between the layers of the aortic wall. The causes may be a penetrating aortic ulcer, an intramural hematoma, or a ruptured thoracic aortic aneurysm. The mortality rate is high, reaching about 1% per hour. If not treated, about 50% of patients are expected to be dead within three days, and almost 80% by the end of the 2nd week [1,2,3,4,5,6]. AAAD is mostly accompanied symptomatically by severe chest or upper back pain of sudden onset. It might also be associated with loss of consciousness or shortness of breath [6,7,8]. Neurological complications are considered life-threatening after AAAD, causing verbal and motoric impairment and, in some cases, can lead to brain death [9,10,11]. The mortality after AAAD with neurological deficits reaches up to 40.2% in patients with cerebrovascular involvement, and 63.0% in patients with coma [12]. Patients presenting with diagnosed AAAD must be surgically operated on with emergency indication. The surgical therapy often includes the replacement of the affected area of the aorta with a vascular prothesis with the help of the heart–lung-machine using moderate hypothermic circulatory arrest (MHCA) [13,14]. The outcome after surgical repair may differ from one patient to another depending on the age, gender, case severity, perioperatively status and intra- or postoperative complications. The aim of this large retrospective study was to evaluate the neurological outcomes in elderly patients who underwent the surgical treatment of AAAD.

## 2. Materials and Methods

### 2.1. Patient Population

All patients with AAAD enrolled in our Registry of AAAD from 2001 to 2016. AAAD is defined as the occurrence of a dissection involving the ascending aorta within 48 h from the first onset of symptoms. In total, retrospectively, 368 consecutive patients who underwent emergency surgery of ascending aorta in MHCA and antegrade cerebral perfusion after AAAD, were analysed. Patients were divided into two groups: patients aged 75 years and older (elderly group; 68 (18.5%)) and patients younger than 75 years (younger group; 300 (81.5%)). The age limit was selected according to Friedrich et al. (2009), that reported more prognosis-determining comorbidities and risk factors in patients over 75 years old with the collaboration of the German Federal Quality Assurance Office (Bundesgeschäftsstelle Qualitätssicherung, BQS) [15]. Patients’ data were collected from our institution’s database and the hospital medical records. The protocol was approved by the local Ethics Committee in Kiel (D417/17). The diagnosis, as well as the exact location, and extension of the dissection membrane, were confirmed preoperatively by a contrast computed tomography (CT). If possible, patients were investigated for neurological deficits at admission. Postoperative manifested neurological complications, including ischemic and hemorrhagic insults, were assessed by the consultant neurologist and proofed by head and neck computer tomography as well as, if required, a CT-angiography for the head and neck arteries. The primary endpoints were 30-day mortality and postoperative neurological events. Secondary endpoints were the perioperative variables, such as the postoperative courses, blood loss, transfusion of blood products, and redo-surgery.

### 2.2. Operative Technique

Patients underwent the surgical repair of AAAD with cardiopulmonary bypass (CPB) in MHCA with a nasopharyngeal temperature between 20 and 24 °C. Cannulation was performed either through cannulation of the femoral artery or the distal ascending aorta. Since 2010, the standard technique for arterial cannulation has been the cannulation of the left ventricle via the right upper pulmonary vein [16]. Cannulation of the femoral vein or the right atrium was used for venous drainage. For myocardial protection, a retrograde injection of cold cardioplegic blood solution was used. A bilateral antegrade cerebral perfusion through a balloon catheter was used in cases of prolonged MHCA lasting more than twenty minutes.

### 2.3. Statistics

The statistical programs SPSS 24.0 (SPSS, Chicago, IL, USA) and Stata 10 SE (StataCorp., College Station, TX, USA) were used for statistical analysis and calculation of the characteristic data. The frequency distribution of the sample data was examined for deviations from the normal distribution using the Kolmogorow–Smirnow–Lilliefors test. The mean ± standard deviation was given for normally distributed, continuous variables. The median and the associated quartiles were given for values that were not normally distributed. Categorical variables were given using the number of affected patients (*n*) and a percentage (%). The Chi-square test and, if necessary, the exact Fisher test were used to compare the two groups examined. All *p*-values ≤ 0.05 were rated as a significant difference between the two groups. A multivariable logistic regression was selected due to clinical relevance and analyzed with backward elimination to determine their relative impact (adjusted odds ratio, OR) on 30-day mortality. Variables included were age >75 years, Euro-SCORE II, coronary heart disease, cardiopulmonary resuscitation, length of surgery (min), cardiopulmonary bypass time (min), MHCA time, number of packed units of red blood cell transfusion, postoperative new-onset of haemodialysis, and postoperative TIA or stroke.

## 3. Results

Comparing both groups, the average age of the patients in our study was 79.0 ± 3.2 vs. 59.2 ± 10.7 years (*p* < 0.001). The female gender represents 58.8% of elderly patients vs. 28.7% in younger patients (*p* < 0.001). Elderly group had a higher significant Euro-score II (9.6% (5.8; 19.2) vs. 4.48% (2.75; 10.45); *p* < 0.001)). In the elderly group, atrial fibrillation prevalence was significantly higher (23.9% vs.11.7; *p* = 0.009). Further significant differences regarding clinical presentations between both groups were not detected (Table 1).

Intraoperatively, our study illustrated that the cardiopulmonary bypass time (155 min (131; 187) vs. 171 min (137; 220); *p* = 0.012) and cross-clamping time (79 min (60; 105) vs. 93 min (71; 134); *p* = 0.001) as well as circulatory arrest time (29 min (22; 40) vs. 33 min (26; 49); *p* = 0.011) were significantly shorter in the elderly group than in the younger group. This is because the surgical procedures performed on the younger group were more complicated (total aortic arch replacement, frozen elephant truck procedures or David procedure) than the one performed on the elderly group. On the other hand, our study has shown that none of the elderly group underwent David operation in comparison to the younger group (0.0% vs. 7.3%; *p* = 0.019). However, our results have shown that the number of young patients who underwent Conduit/ Bental operation as a surgical treatment for AAAD is significantly more than the elderly patients (20% vs. 4.4%; *p* = 0.002). These types of procedure normally need a longer bypass and clamping time, which explains the difference in our results between the two groups (Table 2).

Postoperatively, no significant differences were noticed between both groups regarding postoperative complications. Moreover, the occurrence of postoperative delirium (11.8% vs. 20.5%; *p* = 0.097) or incidence of stroke (11.8% vs. 16.1%; *p* = 0.369) was lower in the elderly group. Thirty-day mortality rate was still satisfactory for the elderly group, but significantly higher compared with the younger group (27.9% vs. 14.3%; *p* = 0.007). A high incidence of neurological deficits occurred in operations longer than 242 min. in older patients. A high incidence of neurological deficits in elderly patients occurred in operations that took longer than 242 minutes. On the other hand, our study has shown that the incidence of neurological deficits in young patients was high in operations that took more than 190 minutes. This could contribute to the aggressive repair in young patients (Table 3).

The multivariate logistic regression confirmed that older age (>75 years), preoperative cardiopulmonary resuscitation (CPR), cardiopulmonary bypass time and postoperative acute kidney injury (AKI) were statistically significant independent risk factors for 30-day mortality (Table 4).

## 4. Discussion

Our study compared the outcomes of 368 consecutive patients who underwent an emergency surgical repair of AAAD, focusing on the risk of the occurrence of postoperative neurological complications in a comparison between elderly patients aged more than 75 years and those younger than 75 years.

One of our main intraoperative findings was that the younger group needed significantly more cardiopulmonary bypass and cross-clamping time than the elderly group. This was contributed to by the prolonged, complicated surgical techniques, such as semi/complete aortic arch replacement or frozen elephant trunk operation, carried out in young patients. These complex surgical procedures need more clamping and bypass time, which puts the patient at risk of postoperatively developing neurological complications. On the other hand, most of the elderly patients underwent simpler surgical procedures, which needed shorter clamping and bypass time.

A study by Mehta et al. focused on the outcome of the management of AAAD in the elderly and collected 550 patients within the elderly (>70 years old) and young (<70 years old) groups [6]. The study showed that the elderly patients developed fewer neurological complications during hospitalization in comparison with the young group. Mehta et al. stated that the reason behind this result may be the involvement of one of the main branches of the aorta, which significantly influences the postoperative neurological deficits in comparison with old patients presenting with a lower incidence of pulse deficits [6]. This result is in proportion with our results, as our elderly group underwent fewer complex surgeries such as aortic arch replacement or re-implantation of the main branches of the head and neck vessels, which also explains why the elderly group suffered less from postoperative neurological deficits than patients younger in age.

Patients who developed strokes in the form of hemiplegia, paraplegia, speech disorder or vision disorder after the surgery were diagnosed using neurological clinical assessment and cranial computer tomography (CT). Patients who developed delirium after surgery were assessed using the confusion assessment method for intensive care unit (CAM-ICU) [17,18]. Postoperative delirium can be expected after surgery by many perioperative factors, such as age, alcohol consumption, cardiopulmonary bypass time, und antegrade selective cerebral perfusion time, and mechanical ventilation time [19]. However, our study results have shown that only 20.5% of the young patients, compared to 11.8% of the elderly patients, experienced delirium, with no significance between both groups after the operation during their admission to the intensive care unit.

Regarding stroke, a postoperative neurological complication is considered one of the most life-threatening complications, whether it is temporary or permanent, causing possible verbal, vision, and movement impairment or restrictions, and in some cases can lead to brain death [9,10,11]. In 2018, a Chinese study from Zhao et al. with a total of 255 patients showed that the postoperative neurological complications which occur after surgical repair to AAAD patients can be determined through CT-angiography before the surgical procedure. In this study, the focus was on the neurological damage as a neurological complication which can occur through a cerebral infarction on top of air or thrombus embolus or due to an acute closure of a supplying artery. The neurological damage in this study was divided into temporary and permanent damage. This study has shown that temporary postoperative neurological damage occurrence (reported 2.5–33%) is correlated with the perioperative CT-angiography findings showing a site of tear entry in the aortic arch or the descending aorta, as well as the dissection of the common carotid artery and unilateral internal carotid artery. On the other hand, it has also shown that permeant postoperative neurological damage occurrence (reported 4.2–24%) is correlated with perioperative CT-angiography findings, showing retrograde dissection of the ascending aorta, an entry tear in the ascending aorta or aortic arch, and low attenuation in the unilateral internal carotid artery [20].

The challenge every surgeon faces with every AAAD surgical repair is avoiding, or at least minimizing, the incidence and the risk factor of the postoperative complications. In 2016, Naito et al. focused, in their study, on the permanent neurological deficit following surgical repair for AAAD, their incidence and the risk factors related to it. In a study with a total 669 patients who underwent emergency surgery by AAAD, 441 patients were treated with ascending/hemiarch replacement operation and 288 patients were treated with total arch replacement operation. The study showed that there is no significant difference in the incidence of permanent neurological damage in the ascending/hemiarch and the total aortic arch replacement. However, perioperative myocardial ischemia and neurological deficits were proven to be risk factors of postoperative permanent neurological damage. In addition, shortening the time between the diagnosis and the start of the surgical treatment, along with minimizing the operation time, is recommended to reduce the incidence of postoperative neurological deficit and obtain better outcomes [21].

A different study from Dumfarth et al., collected the data of 338 patients and discussed the impact of the neurological complications associated with AAAD on the outcome of emergency surgical treatment in comparison with patients who were free from preoperative neurological deficit. A total of 50 patients had preoperative neurological deficit and 288 presented with no neurological deficit. Almost 66 % of the patients who had preoperative neurological deficit had suffered from right hemispheric strokes. The other group presented with bilateral cerebral ischemia. Cranial CT scans were performed with aortic and supraaortic vessel CT scans to preoperatively detect any cerebral ischemia, infarctions or strokes. It was illustrated that the main cause behind the neurological complication associated with AAAD is cerebral perfusion impairment due to occlusion one or more of the supraaortic vessels. The dissection flap or a thromboembolism can be the cause of the occlusion [22].

The International Registry of Acute Dissection (IRAD) reported a critical result in a larger study population of 1873 patients with AAAD regarding the neurological complications and their influence on the mortality rate. The overall hospital mortality in patients without brain injury was 22.7%, 40.2% in patients with cerebrovascular accident, and 63.0% in patients with coma (*p* < 0.001). However, the treatment strategy influenced the mortality rate (76.2% medical vs. 27.0% surgical in cases with cerebrovascular accident; *p* < 0.001 and 100% medical vs. 44.4% surgical in cases with coma; *p* < 0.001) [14].

The previous studies proved that the age of the patient has no direct relation to postoperative neurological complication. Zheng et al., and Stamou et al., reported that old patients (older than 70 years) could safely undergo surgery of AAAD with acceptable rates of mortality and neurological deficits [3,23]. As well, an overall conclusion from the German Registry of Acute Aortic Dissection Type A (GERAAAD) found that the surgical management of AAAD was still associated with significantly lower in-hospital mortality and neurological damage than medical management until the age of 80 years [10]. Rylski B et al., reported, in a study belonging to GERAAAD, a mortality rate of 15.8% in patients between 70 and 80 years old, and 34.9% in patients older than 80 years. The high mortality rate in the older group of patients was attributed to the poorer preoperative condition of octogenarians [24]. Our results also showed no significant difference in the occurrence of stroke as a postoperative complication following AAAD surgical repair in a comparison between both groups regarding age. In spite of that, we recommend avoiding aggressive surgical repair in older patients and reducing the duration of surgery, if possible. This was confirmed in a previous study belonging to our research groups, which found out that the length of surgery and cardiopulmonary bypass time was significantly shorter in survivors after AAAD surgery than those of non-survivors. The strategy of extensive surgical repair, including aortic arch replacement, can only be justified in properly selected patients [25].

## 5. Conclusions

Our study concluded that surgical treatment of AAAD in elderly patients can be applied safely, without increasing risk of neurological complication. The reasons for complications and mortality can be multifactorial. Minimizing the cross-clamping and bypass time, i.e., reducing the operation time may help limit the occurrence of postoperative neurological complication, especially in elderly patients.

## Figures and Tables

**Table 1:** Demographic and clinical characteristics of the study population.

No. of Patients 363	Total	Age < 75 Years	Age ≥ 75 Years	*p*-Value
No. of surgical procedures	368 (100%)	300 (81.5%)	68 (18.5%)	
Age, years	62.8 ± 12.4 63.7 (54.2; 72.5)	59.2 ± 10.7 60.4 (52.2; 68.1)	79.0 ± 3.2 78.2 (76.1; 82.4)	<0.001
Female Gender	126 (34.2%)	86 (28.7%)	40 (58.8%)	<0.001
EuroSCORE II (%)	5.45 (3.06; 12.41)	4.48 (2.75; 10.45)	9.6 (5.8; 19.2)	<0.001
Coronary heart disease	63 (18.0%)	51 (17.8%)	12 (18.8%)	0.863
Previouspercutaneous coronary intervention (+/−Drug-eluting stent)	24 (6.8%)	19 (6.6%)	5 (7.7%)	0.784
Previous thoracic surgery	40 (11.1%)	33 (11.3%)	7 (10.6%)	0.878
Previous coronary artery bypass grafting	12 (3.4%)	9 (3.1%)	3 (4.6%)	0.464
Peripheral vascular disease	16 (4.5%)	12 (4.2%)	4 (6.2%)	0.508
Left ventricular ejection fraction (%)	64 (55; 70)	65 (55; 70)	60 (50; 70)	0.266
Marfan syndrome	9 (2.5%)	8 (2.7%)	1 (1.5%)	1.000
Aortic aneurysm	113 (30.8%)	90 (30.1%)	23 (33.8%)	0.548
Diameter of aneurysm, mm	53 ± 12	53.4 ± 12.9	52.7 ± 9.8	0.830
Calcific aortic disease	9 (2.5%)	5 (1.7%)	4 (6.0%)	0.063
Bicuspid aortic valve	9 (2.4%)	8 (2.7%)	1 (1.5%)	1.000
Aortic valve				
Aortic valve intact	168 (53.2%)	141 (53.8%)	27 (50.0%)	
Aortic valve stenosis	10 (3.2%)	5 (1.9%)	5 (9.3%)	
Aortic valve insufficiency	130 (41.1%)	109 (41.6%)	21 (38.9%)	
Combined Aortic valve vitium	8 (2.5%)	7 (2.7%)	1 (1.9%)	
Neurological deficits	73 (20.3%)	57 (19.5%)	16 (23.9%)	0.424
Clinical presentation				
Acute myocardial infarction (48h)	14 (3.8%)	12 (4.0%)	2 (3.0%)	1.000
Cardiogenic shock	27 (7.4%)	19 (6.4%)	8 (12.1%)	0.121
Cardiopulmonary resuscitation	31 (8.5%)	22 (7.4%)	9 (13.6%)	0.100
Urgency of admission				
Elective	49 (13.3%)	43 (14.3%)	6 (8.8%)	
Urgent	301 (81.8%)	242 (80.7%)	59 (86.8%)	
Emergency	18 (4.9%)	15 (5.0%)	3 (4.4%)	

**Table 2:** Intraoperative data.

	Total	Age < 75 Years	Age ≥ 75 Years	*p*-Value
Length of surgery, min	280 (227; 345)	285 (229; 349)	253 (217; 304)	0.011
Cardiopulmonary bypass time, min	166 (135; 212)	171 (137; 220)	155 (131; 187)	0.012
Cross-clamp time, min	89 (70; 127)	93 (71; 134)	79 (60; 105)	0.001
Circulatory arrest, min	33 (26; 46)	33 (26; 49)	29 (22; 40)	0.011
Antegrade cerebral perfusion (n)	162 (44%)	140 (46.6%)	22 (32%)	1.000
Number of packed red blood cells	4 (0; 6)	3 (0; 6)	4 (2; 8)	0.004
Number of fresh frozen plasma	0 (0; 6)	0 (0; 6)	2 (0; 4.5)	0.326
Number of platelets concentrate	2 (1; 2)	2 (1; 2)	2 (1; 2)	0.358
Surgical procedure				
Isolated supracoronary ascending aortareplacement	186 (50.5%)	143 (47.7%)	43 (63.2%)	0.020
Partial aortic arch replacement	78 (21.2%)	62 (20.7%)	16 (23.5%)	0.602
Total aortic arch replacement	48 (13.0%)	42 (14.0%)	6 (8.8%)	0.252
Conduit/Bentall operation	63 (17.1%)	60 (20.0%)	3 (4.4%)	0.002
David operation	22 (6.0%)	22 (7.3%)	0 (0.0%)	0.019
Elephant-trunk	7 (1.9%)	7 (2.3%)	0 (0.0%)	0.357
CABG	35 (9.6%)	28 (9.5%)	7 (10.3%)	0.833
Aortic valve replacement	57 (15.5%)	49 (16.4%)	8 (11.8%)	0.342
Mitral valve reconstruction/replacement	1 (0.3%)	1 (0.3%)	0 (0.0%)	1.000
Tricuspid valve reconstruction/replacement	0 (0.0%)	0 (0.0%)	0 (0.0%)	
Patent foramen ovale closure	5 (1.4%)	5 (1.7%)	0 (0.0%)	0.589
TEVAR(EVAR)	23 (6.3%)	20 (6.7%)	3 (4.4%)	0.591
Arterial cannulation				
Femoral artery	74 (22.2%)	63 (23.2%)	11 (17.7%)	
Ascending aorta	85 (25.4%)	68 (25.0%)	17 (27.4%)	
Aortic arch	14 (4.2%)	9 (3.3%)	5 (8.1%)	
Subclavian artery	1 (0.3%)	1 (0.4%)	0 (0.0%)	
Apex	5 (1.5%)	5 (1.8%)	0 (0.0%)	
Pulmonary vein	155 (46.4%)	126 (46.3%)	29 (46.8%)	
Venous cannulation				
Right atrium	322 (96.4%)	262 (96.3%)	60 (96.8%)	
Bicaval	4 (1.2%)	4 (1.5%)	0 (0.0%)	
Femoral vein	8 (2.4%)	6 (2.2%)	2 (3.2%)	

**Table 3:** Postoperative data and outcomes.

	Total	Age < 75 Years	Age ≥ 75 Years	*p*-Value
AKI KDIGO	80 (22.0%)	63 (21.3%)	17 (25.4%)	0.466
New—onset of Hemodialysis	80 (21.9%)	62 (20.8%)	18 (26.9%)	0.279
Temporary dialysis	4 (2; 12)	4 (2.5; 12.5)	3.5 (1.0; 5.3)	0.156
48 h-drainage loss, mL	825 (450; 1250)	800 (500; 1300)	850 (350; 1138)	0.187
Postoperative blood transfusion	247 (70.8%)	205 (71.2%)	42 (68.9%)	0.716
Total number of packed red blood cells	3 (0; 7)	2 (0; 7)	3 (0; 7.5)	0.789
Total number of fresh frozen plasma	0 (0; 4)	0 (0; 4)	0 (0; 4.5)	0.801
Total number of platelets concentrate	0 (0; 1)	0 (0; 2)	0 (0; 1)	0.202
Reintubation	66 (18.0%)	58 (19.4%)	8 (11.8%)	0.139
Tracheotomy	84 (22.9%)	70 (23.4%)	14 (20.6%)	0.617
Re-admission to the ICU	33 (9.0%)	26 (8.7%)	7 (10.4%)	0.651
Re-admission (postoperative days)	5.6 ± 4.5	5.1 ± 4.3	6.7 ± 5.1	0.205
Postoperative delirium	69 (18.9%)	61 (20.5%)	8 (11.8%)	0.096
Postoperative myocardial infarction	4 (1.1%)	4 (1.3%)	0 (0.0%)	1.000
Transient ischemic attack/Stroke (CT scan-proofed)	56 (15.3%)	48 (16.1%)	8 (11.8%)	0.369
Relation of high incidence of neurological deficits to operation time	>216 min	>190 min	>242 min	
Electrical cardioversion	26 (7.1%)	20 (6.7%)	6 (8.8%)	0.600
Cardiopulmonary resuscitation	28 (7.7%)	23 (7.7%)	5 (7.4%)	0.913
Bronchopulmonary infection	48 (13.1%)	41 (13.8%)	7 (10.3%)	0.445
Bacteriaemia/sepsis	17 (4.6%)	12 (4.0%)	5 (7.4%)	0.333
Rethoracotomy	62 (16.9%)	53 (17.7%)	9 (13.2%)	0.372
Sternal wound infection/VAC revision	6 (1.6%)	5 (1.7%)	1 (1.5%)	1.000
Sinus rhythm	271 (77.0%)	228 (79.2%)	43 (67.2%)	0.039
Atrial fibrillation	40 (11.4%)	32 (11.1%)	8 (12.5%)	0.751
Other Rhythm	10 (2.8%)	8 (2.8%)	2 (3.1%)	1.000
Pacemaker patient	21 (5.8%)	16 (5.4%)	5 (7.5%)	0.560
Ventilation time, h	64 (21; 188)	60 (20; 196)	87 (30; 173)	0.284
ICU time, days	5 (2; 11)	5 (2; 12)	5 (3; 9)	0.685
Postoperative days	11 (7; 19)	11 (8; 20)	9 (7; 14)	0.022
Mortality POD	2.5 (1; 8)	2 (1; 8)	3.5 (1; 9.3)	0.778
7 d-Mortality	47 (13.1%)	32 (10.9%)	15 (22.7%)	0.010
30 d-Mortality	62 (18.0%)	43 (14.3%)	19 (27.9%)	0.007
Hospital Mortality	61 (17.0%)	42 (14.4%)	19 (28.4%)	0.006
Cardiac death	36 (56.3%)	23 (52.3%)	13 (65.0%)	
Cerebral death	4 (6.3%)	3 (6.8%)	1 (5.0%)	
Sepsis	3 (4.7%)	2 (4.5%)	1 (5.0%)	
Multi-organ failure	21 (32.8%)	16 (36.4%)	5 (25.0%)	

**Table 4:** Risk factors for the 30-d-mortality.

	Odds Ratio	CI	*p*-Value
Age > 75 years	3.194	1.133–9.005	0.028
CPR	5.111	1.626–16.069	0.005
Bypass time	1.010	1.004–1.015	0.001
AKI KDIGO	10.134	4.412–23.274	<0.001

## Data Availability

Data is available upon requirement.
